# ID 224 - Hemangioma Infantil no Brasil: perfil epidemiológico da mortalidade e dos procedimentos ambulatoriais e hospitalares no Sistema Único de Saúde

**DOI:** 10.5327/2237-9622.2025.v34s1.224

**Published:** 2025-11-25

**Authors:** Nadya Lie Fattori, Ísis Nalin Fernandes Nonato, Gabrielle Ferrante Alves de Moraes, Adriane Lopes Medeiros Simone, Daniela de Oliveira de Melo, Ana Laura de Sene Amâncio Zara

**Keywords:** hemangioma na infância, neoplasias de tecido vascular, estudos de séries temporais, estudos ecológicos

## Abstract

**Introdução:**

Hemangioma infantil (HI) é um dos tumores vasculares benignos mais comuns na infância, acometendo aproximadamente de 4% a 5% da população mundial. Cerca de 80% das lesões de HI são diagnosticadas por meio de exames físicos no primeiro mês de vida, sendo mais frequentes no sexo feminino. O presente estudo teve por objetivo analisar se houve redução na taxa de mortalidade (TM) e nos atendimentos ambulatoriais e hospitalares por HI no Brasil.

**Método:**

Realizou-se um estudo ecológico com dados do Sistema de Informações sobre Mortalidade (SIM), do Sistema de Informações Ambulatoriais do Sistema Único de Saúde (SIA/SUS) e do Sistema de Informações Hospitalares do Sistema Único de Saúde (SIH/SUS), ocorridos em crianças menores de 5 anos de idade. Foram selecionados apenas os casos com código da Classificação Internacional de Doenças (CID-10) D18.0 (hemangioma em qualquer localização). A TM por HI foi estimada dividindo-se o número de óbitos em menores de 5 anos pelo número de nascidos vivos (NV), multiplicado por 1 milhão a cada ano, no período de 2000 a 2022. Utilizando-se o programa Stata, analisou-se a série temporal da TM pelo método de regressão de Prais-Winsten (p<0,05). A taxa incremental média anual (TIMA) foi calculada em porcentagem, com seu respectivo intervalo de confiança de 95% (IC 95%). Realizou-se uma análise descritiva dos procedimentos ambulatoriais e hospitalares (2008 a 2022) e foram estimados seus custos financeiros, corrigidos pela inflação brasileira (IPC-A) em julho de 2024.

**Resultados:**

Foram registrados 145 óbitos por HI em crianças menores de 5 anos, variando de 1,56/1.000.000 NV (2000) a 0,37/1.000.000 NV (2022), com uma redução média de 5,4% ao ano (IC 95%: 9,05; 1,65; p=0,007). De 2008 a 2022, foram registrados 7.392 procedimentos ambulatoriais (493 atendimentos/ano); observou-se que 69% dos atendimentos foram realizados em crianças com menos de 1 ano de idade e 64% no sexo feminino. As intervenções ambulatoriais mais dispendiosas incluem: dispensação de alfainterferona, ultrassonografia de abdômen total e consulta em Atenção Especializada. Foram registrados 25.048 procedimentos hospitalares (1.089 atendimentos/ano) de 2008 a 2022, com 55% das internações em menores de 1 ano e 52% no sexo masculino; verificou-se que as intervenções cirúrgicas foram as mais dispendiosas. Em relação aos custos financeiros do tratamento do HI em crianças menores de 5 anos, o custo médio mensal foi de R$ 85.953,65.

**Conclusão:**

A TM por HI está em queda no Brasil. Há um padrão de atendimentos ambulatoriais e hospitalizações no SUS, em que o principal público atendido foi de crianças menores de 1 ano de idade e do sexo feminino, reforçando os dados epidemiológicos encontrados na literatura. Houve aumento nos atendimentos ambulatoriais a partir de 2018, possivelmente associado à publicação do Protocolo Clínico e Diretriz Terapêutica para HI. Isso corrobora a redução nas hospitalizações e mostra que diagnóstico, o acompanhamento e as orientações precoces podem evitar a evolução da doença, sendo esse um importante fator na construção de políticas públicas voltadas para o tratamento do HI.

